# Is there a future for hysteroscopic sterilisation?

**DOI:** 10.52054/FVVO.16.4.053

**Published:** 2024-12-17

**Authors:** G Chene

**Affiliations:** Department of Gynecology, Hôpital Femme Mère Enfant, HFME, 59 Boulevard Pinel, University Hospital of Lyon, 69500 Bron, Franc; University Claude Bernard of Lyon 1, EMR 3738 CICLY, 69000 Lyon, France

**Keywords:** Essure®, hysteroscopic sterilization, laparoscopic sterilization, Adiana®, Ovabloc®, AltaSeal®, FemBloc®

When novel hysteroscopic sterilisation techniques were introduced in the 2000s, it was heralded as the beginning of the end of laparoscopic approaches. Hysteroscopic techniques were considered less invasive, avoiding incisions and scars, technically easier and safer without risk of major complications. Moreover, the hysteroscopic route was more convenient for women because the procedure could be performed under local anaesthesia in an outpatient setting. However, once the technologies were introduced into general gynaecological practice concerns were raised about discrepancies in the rates of successful bilateral tubal occlusion and how to best confirm the correct placement of the devices, and the pregnancy risk after correcting bilateral placement. Over time, increasing reports of adverse events and chronic symptoms are thought to be related to the presence of the micro-inserts accumulated. Taken all together these concerns led to the withdrawal of the new hysteroscopic sterilisation technologies by the manufacturers.

In this issue of Facts, Views and Vision, van Gastel et al. (2024) present the long-term results of Adiana® (Hologic©, USA) hysteroscopic tubal occlusion in the Netherlands. The Adiana® procedure involved the application of bipolar radiofrequency energy to create a thermal tubal injury followed by the placement of the non-biodegradable porous implant. The authors found bilateral tubal occlusion confirmed by hysterosalpingography in 77.1% of participants and an unacceptable pregnancy rate of 3.6%. A meta- analysis about the effectiveness of three hysteroscopic sterilisation techniques in 2015 (La Chapelle et al., 2015) found a higher 78%-98% probability of successful bilateral device placement at the first attempt (respectively, 78%-84% for Ovabloc® device (Advanced Medical Grade Silicones BV©, The Netherlands), 81-98% for Essure® (Bayer©, Germany) and 94% for Adiana®). The pregnancy rates of the three hysteroscopic tubal occlusion devices after correct placement were lower than observed in the series reported in this issue of FVVO (van Gastel et al., 2024); 1% for Ovabloc®, 0.1% for Essure® and 1.6% for Adiana®) (La Chapelle et al., 2015; Anderson and Vancaillie, 2011).

Ovabloc® was first withdrawn from the market in 2009 because of suboptimal clinical results and technical difficulties with the cold storage of the silicon. Adiana® was withdrawn in 2012 after an infringement litigation between the companies Conceptus (Essure®) and Hologic (Adiana®). Though the Essure® permanent birth control system was then the only remaining hysteroscopic sterilisation technology left for sterilisation, the marketing of Essure® implants was definitively stopped by Bayer© in 2018 after around 900,000 procedures worldwide for commercial reasons.

Since 2015 many patients complained of gynaecological symptoms after Essure® sterilisation such as heavy bleeding and pelvic pain and also non-specific systemic symptoms such as tiredness, hair loss, alopecia, depression, loss of libido, painful joints, weight changes and lack of concentration (Insubri et al., 2024; Parant et al., 2022; Maassen et al., 2019). Evidence is lacking about any relationship between Essure® and these supposed adverse events such that biological and clinical studies are needed (Chene and Graesslin, 2022). Despite this lack of understanding, several pathophysiological hypotheses have been provided (Parant et al., 2022):

(i) Nickel allergy or sensitivity (nickel is one of the main constituents of the Essure® implants).
(ii) The possible galvanic corrosion of Essure® and the concomitant release of heavy, potentially toxic, metals since several studies have shown a dynamic dissemination of certain metallic elements in the Fallopian tubes as well as outside the tubes.
(iii) The inflammation theory, alone or together with other biological mechanisms. Essure® micro-coils contain polyethylene terephthalate (PET) fibres, which are known to create a moderate foreign-body inflammatory process.

So, with the apparent failure of hysteroscopic sterilisation is male sterilisation set to be the preferred sterilisation method? Whilst there has been an increase in vasectomy rates seen in many countries, female sterilisation by the abdominal or laparoscopic route remains the world’s most widely used permanent contraceptive method (Jacobstein et al., 2023).

Thus, there appears to remain an unmet need for a less invasive female method of birth control despite the availability of alternative effective contraceptives like the levonorgesterol-releasing intrauterine system (LNG-IUS) or etonogestrel subdermal implant. However, will it ever be possible to develop a hysteroscopic or other intrauterine, non-incisional method of female sterilisation? Hysteroscopic sterilisation is associated with a significantly lower risk of surgical complications than laparoscopic sterilisation (adjusted OR, 0.18; 95% CI, 0.14 to 0.23; adjusted RD, −0.64; 95% CI, −0.67 to −0.60) and medical (adjusted OR, 0.51; 95% CI, 0.30 to 0.89; adjusted RD, −0.05; 95% CI, −0.08 to −0.01) complications of the sterilisation procedure (Bouillon et al., 2018). Hysteroscopic sterilisation performed under local anaesthesia is associated with shorter hospital stay and more rapid recovery (La Chapelle et al., 2015). Given these advantages, we should remain determined and optimistic, as the writer William R Alger stated, “After every storm, the sun will smile; for every problem, there is a solution, and the soul’s indefeasible duty is to be of good cheer.”

There is ongoing research into a new hysteroscopic sterilisation device, AltaSeal® (AltaScience©, Ireland), a biocompatible 316LVM stainless steel device that provides mechanical tubal occlusion (Coleman et al., 2017). Another non-incisional, implant-free method of female sterilisation under clinical evaluation is, FemBloc® (Femasys Inc.©, GA, US) (NCT05977751). This technology does not require hysteroscopic guidance and involves the outpatient delivery of a biopolymer into both Fallopian tubes that solidifies and then degrades and expels within three months. During this time tissue in-growth is promoted to provide permanent tubal blockage, confirmed using ultrasound.

Lessons should be learned from the problems with previous sterilisation hysteroscopic technologies and inform future innovation and technological development. The “ideal hysteroscopic sterilisation method” should allow storage under room temperature, provide minimally invasive and immediate mechanical occlusion of the fallopian tubes, and need no confirmation testing or at most imaging that avoids ionising non- radiation diagnostic tests to confirm the correct placement (e.g. two or three- dimensional ultrasound instead of hysterosalpingography or pelvic radiography) (La Chapelle et al., 2015). There is also an imperative need for RCTs, cohort studies with long-term post-marketing follow-up as well as complete biological evaluation studies (to look for corrosion characteristics and cellular behaviour of the biomaterials) to confirm surgical feasibility, effectiveness and lack of adverse events and complications.
